# Pulmonary vascular permeability index and global end-diastolic volume: are the data consistent in patients with femoral venous access for transpulmonary thermodilution: a prospective observational study

**DOI:** 10.1186/1471-2253-14-81

**Published:** 2014-09-24

**Authors:** Helena Berbara, Sebastian Mair, Analena Beitz, Benedikt Henschel, Roland M Schmid, Wolfgang Huber

**Affiliations:** 1 II. Medizinische Klinik und Poliklinik, Klinikum rechts der Isar der Technischen Universität München, Ismaninger Straße 22, D-81675 München, Germany

**Keywords:** Lung edema, ARDS, Extravascular lung water, Pulmonary vascular permeability index, Transpulmonary thermodilution, Femoral venous catheter, Hemodynamic monitoring, Cardiac output, Catheter site, PiCCO

## Abstract

**Background:**

Transpulmonary thermodilution (TPTD) derived parameters are used to direct fluid management in ICU-patients. Extravascular lung water EVLW and its ratio to pulmonary blood volume (pulmonary vascular permeability index PVPI) have been associated with mortality. In single indicator TPTD pulmonary blood volume (PBV) is estimated to be 25% of global end-diastolic volume (GEDV). A recent study demonstrated marked overestimation of GEDV indexed to body-surface area (BSA; GEDVI) when using a femoral central venous catheter (CVC) for indicator injection due to the additional volume measured in the vena cava inferior. Therefore, a correction formula derived from femoral TPTD and biometric data has been suggested. Consequence, one of the commercially available TPTD-devices (PiCCO; Pulsion Medical Systems, Germany) requires information about CVC site. Correction of GEDVI for femoral CVC can be assumed. However, there is no data if correction also pertains to unindexed GEDV, which is used for calculation of PBV and PVPI. Therefore, we investigated, if also GEDV, PBV and PVPI are corrected by the new PiCCO-algorithm.

**Methods:**

In this prospective study 110 triplicate TPTDs were performed within 30 hours in 11 adult ICU-patients with PiCCO-monitoring and femoral CVC. We analyzed if the femoral TPTD correction formula for GEDVI was also applied to correct GEDV. Furthermore, we compared PVPI_displayed_ to PVPI_calculated_ which was calculated as EVLW_displayed_/(0.25*GEDV_displayed_).

**Results:**

Multiplication of GEDVI_displayed_ by BSA resulted in GEDV_calculated_ which was not significantly different to GEDV_displayed_ (1459 ± 365 mL vs. 1459 ± 366 mL) suggesting that correction for femoral indicator injection also pertains to GEDV_displayed_. However, PVPI_displayed_ was significantly lower than PVPI_calculated_ (1.64 ± 0.57 vs. 2.27 ± 0.72; p < 0.001). In addition to a bias of -0.64 ± 0.22 there was a percentage error of 22%. Application of the correction formula suggested for GEDVI to PVPI_displayed_ reduced the bias of PVPI_displayed_ compared to EVLW/PBV from -0.64 ± 0.22 to -0.10 ± 0.05 and the percentage error from 22% to 4%.

**Conclusions:**

Correction for femoral CVC in the PiCCO-device pertains to both GEDVI_displayed_ and GEDV_displayed_, but not to PVPI_displayed_. To provide consistent information, PVPI should be calculated based on GEDV_corrected_ in case of femoral CVC.

## Background

Appropriate fluid supply is a cornerstone of critical care. In addition to cardiac output CO, transpulmonary thermodilution (TPTD) provides extravascular lung water (EVLW) reflecting pulmonary edema and the preload parameter global end-diastolic volume (GEDV) [1-6]. Increased EVLW is associated to mortality [3,7-11]. GEDV and its changes are associated to CO [6] and algorithms based on GEDV have been shown to improve outcome [12]. Moreover, the relation of EVLW and pulmonary blood volume (PBV) might be useful to differentiate the etiology of pulmonary edema [13,14]. PBV can be determined using double-indicator TPTD technique [13]. Single indicator TPTD is much more common in clinical routine and estimates PBV as about 25% of GEDV [14]. EVLW/PBV-ratio has been termed pulmonary vascular permeability index “PVPI”. Several studies demonstrated that higher values of PVPI are associated to pulmonary edema with increased permeability of the alveolo-capillary barrier [14]. Increased PVPI has been found in patients with ARDS resulting from pulmonary (e.g. pneumonia) or secondary (e.g. sepsis) pulmonary impairment. By contrast hydrostatic pulmonary edema due to congestive heart failure usually does not result in marked increases of PVPI since EVLW and PBV are increased to a similar extent [13,14].

**Figure 1:**
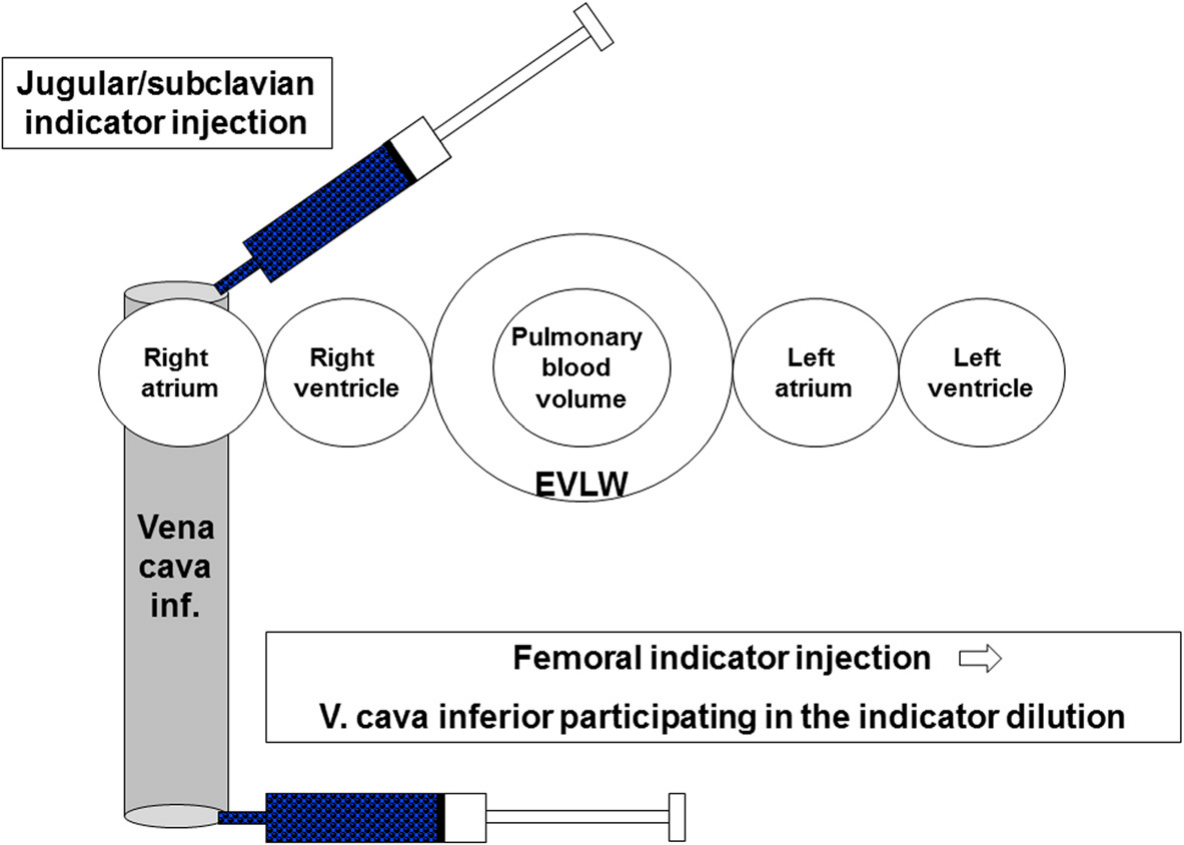
**Comparison of transpulmonary indicator dilution (TPTD) using femoral central venous catheter (CVC) instead of jugular or subclavian CVC.** Obviously, the distribution volume for the indicator is augmented by the volume of vena cava inferior in case of femoral indicator injection. This results in diminished indicator concentration and overestimation of global enddiastolic volume GEDV. In single indicator dilution technique GEDV is calculated as difference of the total dilution volume (intrathoracic thermovolume ITTV) minus the sum of pulmonary blood volume PBV and extravascular lung water EVLW: GEDV = ITTV- (EVLW + PBV). The sum (PBV + EVLW) is thermed pulmonary thermovolume PTV, which is directly derived from the thermodilution curve. Therefore, it can be assumed not to be altered by the additional dilution volume of VCI participating in TPTD.

Although TPTD has become part of clinical routine, several pitfalls have to be kept in mind. Two recent studies demonstrated marked overestimation of GEDV in case of performing TPTD indicator injection using a femoral venous access due to the additional volume of vena cava inferior (VCI) participating in TPTD [15,16]; Figure 1).

The authors suggested a correction formula for GEDV indexed to predicted body surface area (GEDVI) based on data derived from femoral TPTD and biometric information. As a consequence of these data one the manufacturer of the PiCCO device (Pulsion Medical Systems, Feldkirchen, Germany) implemented a new software requiring the information about CVC site (femoral or jugular/subclavian) and correcting GEDVI in case of femoral injection. Although jugular and subclavian vein access might be the preferred sites for CVC insertion, several recent studies on catheter related bloodstream infections have demonstrated that femoral venous access was used in about 20 to 35% of all catheter insertions [17,18]. Therefore, results of TPTD might be substantially altered in about one third of cases without appropriate correction.

To the best of our knowledge there are no data available if the new PiCCO algorithm also corrects unindexed GEDV and PVPI in case of femoral CVC.

Furthermore, data on PVPI were not investigated in both previous papers on TPTD using a femoral CVC [15,16].

Therefore, it was the aim of our study to investigate if in case of femoral indicator injection

GEDV in the new PiCCO algorithm is given based on the correction of GEDVI and

PVPI is based on corrected GEDV and corrected pulmonary blood volume.

## Methods

The study was approved by the institutional review board (Ethikkommission der Fakultät für Medizin der Technischen Universität München, Munich, Germany).

All patients or their legal representatives gave written informed consent.

We prospectively performed 110 TPTDs in 11 patients with femoral indicator injection. All patients were recruited between October 2013 and January 2014.

Ten triplicate TPTDs per patient were performed with 15 mL cold saline within a total of 30 h. TPTD was performed as described previously using the PiCCO-2 device (Pulsion Medical Systems, Feldkirchen, Germany) [6,16,19] and a 5-lumen CVC (Multicath 5; Vygon; Aachen, Germany). All PiCCO-2 devices were equipped with the V3.1 algorithm requiring information about the venous catheter site which was set to “femoral” CVC in all patients.

Immediately after triplicate TPTD absolute and indexed mean values were documented for GEDV, GEDVI, EVLW, EVLWI and PVPI as displayed by the device. Parameters displayed by the device were subscripted with “displayed” (see Table 1).

**Table 1:** Terminology of thermodilution-derived parameters

**Subscript**	**Meaning**	**Parameters analyzed**
“displayed”	Values as displayed by the PiCCO monitor	GEDVI_displayed_
		GEDV_displayed_
		EVLW_displayed_
		PVPI_displayed_
“calculated”	Parameters calculated based on parameters displayed on the monitor.	GEDV_calculated_
	These parameters should be consistent with corresponding displayed values.	PVPI_calculated_
“corrected”	Parameters derived from correction of the displayed values. Corrected parameters were used for comparison to calculated values (only used in case of inconsistency of displayed and calculated values).	PVPI_corrected_
		GEDV_uncorrected_

The main purpose of the study was to investigate the consistency of displayed values by comparing displayed data (e.g. GEDV_displayed_, PVPI_displayed_) to the corresponding values derived from calculation specified with the subscript “calculated” (GEDV_caculated_, PVPI_calculated_).

If displayed and calculated values were inconsistent, we investigated whether the inconsistency could be explained by (non-) application of the recently suggested correction formula for the displayed values parameters [16]. In this case “ex post” correction by our formula should result in a consistency of the “corrected” values (with the subscript “corrected”) and the calculated values.

To analyze if the femoral TPTD correction formula for GEDVI_displayed_ was only applied for GEDVI_displayed_ or if also GEDV_displayed_ is corrected in case of femoral indicator injection, we calculated GEDV_calculated_ using the formula:

$${\mathrm{GEDV}}_{\mathrm{calculated}}\;=\;{\mathrm{GEDVI}}_{\mathrm{displayed}}*{\mathrm{BSA}}_{\mathrm{predicted}}\text{.}$$

Finally, GEDV_displayed_ was compared to GEDV_calculated_.

In a second step we analyzed, if PVPI_displayed_ is calculated based on a GEDV_displayed_ corrected for femoral indicator injection. Therefore, we compared PVPI_displayed_ to PVPI_calculated_ using the formula PVPI_calculated_ = EVLW_displayed_/(0.25 * GEDV_displayed_).

Predicted bodyweight BW_predicted_ was calculated using the formula:

$$\begin{array}{l} \mathrm{Predicted}\;\mathrm{bodyweight}\;{\mathrm{BW}}_{\mathrm{predicted}}\left[\mathrm{kg}\right]:\;\mathrm{Male}:\;50\;+\;0.91\;*\;\left(\mathrm{height}\;–\;152.4\right)\\ \mathrm{Female}:\;45.5\;+\;0.91\;*\;\left(\mathrm{height}\;–\;152.4\right) \end{array}$$

Predicted body surface area BSA_predicted_ was calculated using BW_predicted_ instead of actual bodyweight in the Dubois formula:

$${\mathrm{BSA}}_{\mathrm{Dubois}}\left[m^{2}\right]\;=\;0.007184*\mathrm{weight}\;{\left[\mathrm{kg}\right]}^{0.425}*\mathrm{height}\;{\left[\mathrm{cm}\right]}^{0.725}$$

The correction formula suggested for correction of uncorrected femoral indicator injection derived GEDV_uncorrected_ is GEDVI_corrected_ [mL / m^2^] = 0.539 * GEDVI_uncorrected_ - 15.17 + 24.49 * CI_uncorrected_ +2.311* BW_ideal_[16].

Ideal bodyweight BW_ideal_ was calculated using the formula:

$$\begin{array}{l} \mathrm{Ideal}\;{\mathrm{bodyweightBW}}_{\mathrm{ideal}}\left[\mathrm{kg}\right]:\;\mathrm{Male}:\;\left(\mathrm{height}\;–\;100\right)\;x\;0.9\\ \mathrm{Female}:\;\left(\mathrm{height}\;–\;100\right)\;x\;0.85 \end{array}$$

Correlation of GEDV_calculated_ and GEDVI_displayed_ was analyzed using Spearman correlation. Comparisons of GEDV_displayed_ vs. GEDV_calculated_, PVPI_displayed_ vs. PVPI_calculated_ and PVPI_corrected_ vs. PVPI_calculated_ were performed using Wilcoxon-test for paired samples, respectively. Bland-Altman analyses and calculation of percentage error (PE) were used for these comparisons as described previously [20,21]. Bland-Altman analyses were corrected for repeated measurements allowing variability of true values within each subject [22].

All analyses were performed using IBM SPSS Statistics 21 (SPSS inc., Chicago, IL, USA).

## Results

### Patients characteristics

Five female and six male patients were included. Patients characteristics are shown in Table 2.

**Table 2:** Patients characteristics (mean ± standard deviation; numbers and percentages)

**Gender**	**5/11 (45%) female; 6/11 (55%)male**
Age [years]	59.9 ± 11.9
Height [cm]	171.1 ± 11.7
APACHE-II score	16 ± 6
SOFA score	6.5 ± 2.5
Actual bodyweight [kg]	72.8 ± 19.1
Predicted bodyweight [kg]	65.0 ± 12.0
Ideal bodyweight [kg]	62.5 ± 11.4
Predicted body surface area [m^2^]	1.76 ± 0.22
Aetiology	
Sepsis	7/11 (64%)
ARDS	1/11 (9%)
Cirrhosis	1/11 (9%)
Cardiogenic shock	2/11 (18%)
Measurements under mechanical ventilation	60/110 (54.5%)
Measurements under vasopressors	34/110 (30.9%)

**Figure 2:**
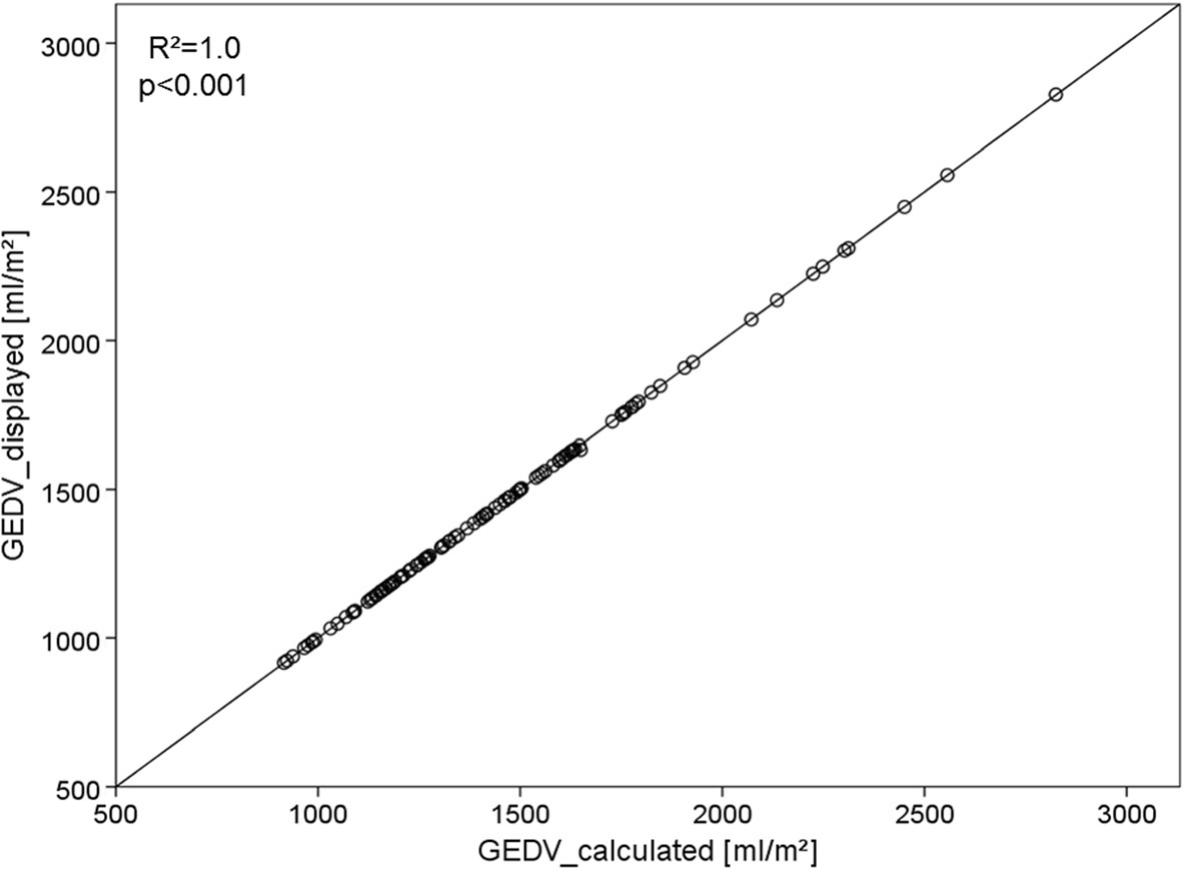
**Scatter plot depicting the correlation of GEDV as displayed by the PiCCO-2 device (GEDV**_**displayed**_**) vs. GEDV calculated as product GEDVI**_displayed _* predicted body surface area.

GEDV_calculated_ was obtained by multiplying GEDVI_displayed_ by BSA_predicted_. As demonstrated in Figure 2 GEDV_calculated_ and GEDV_displayed_ significantly correlated (r^2^ = 1.0; p < 0.001) and were not significantly different (1459 ± 365 mL vs. 1459 ± 366 mL).

This confirms that correction for femoral venous catheter site pertains to both GEDVI_displayed_ and GEDV_displayed_.

Consequently, calculation of PVPI_calculated_ based on EVLW_displayed_ and GEDV_displayed_ should result in a PVPI_calculated_ identical with PVPI_displayed_.

**Figure 3:**
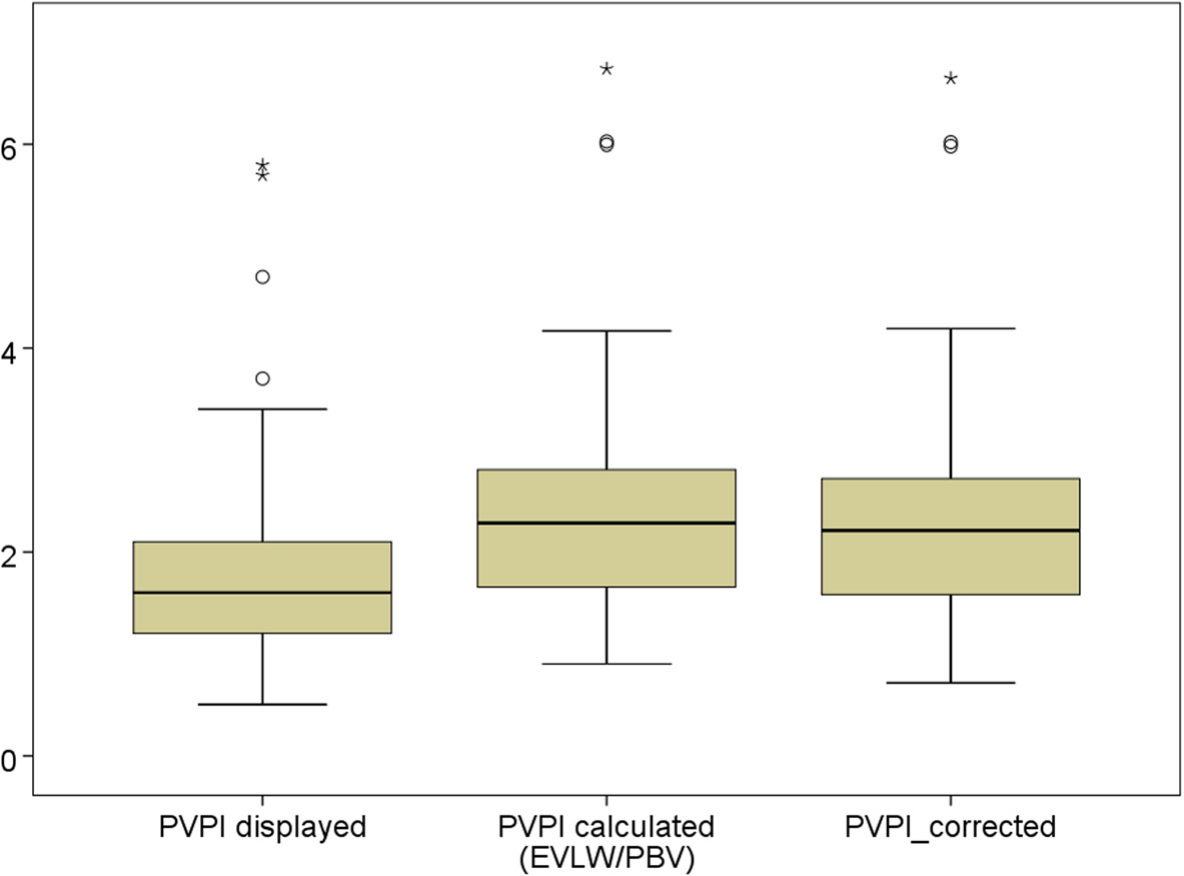
**Boxplots plots comparing pulmonary vascular permeability index PVPI as displayed by the PiCCO-2 device (PVPI**_**displayed**_**) vs. PVPI calculated as ratio of EVLW/PBV (extravascular lung water/pulmonary blood volume) vs. PVPI corrected using the formula suggested for correction of femoral indicator injection derived GEDV **[16]**: GEDVI**_**corrected **_**[mL / m**^**2**^] = 0.539 * GEDVI_**uncorrected **_- 15.17 + 24.49 * CI_**uncorrected **_2.311* BW_**ideal**_**.** Assuming that PVPI_displayed_ was calculated based on GEDV and PBV not corrected for femoral injection, PVPI_corrected_ was calculated by multiplying PVPI_displayed_ with the ratio 0.25*GEDV_uncorrected_/0.25*GEDV_corrected_.

**Figure 4:**
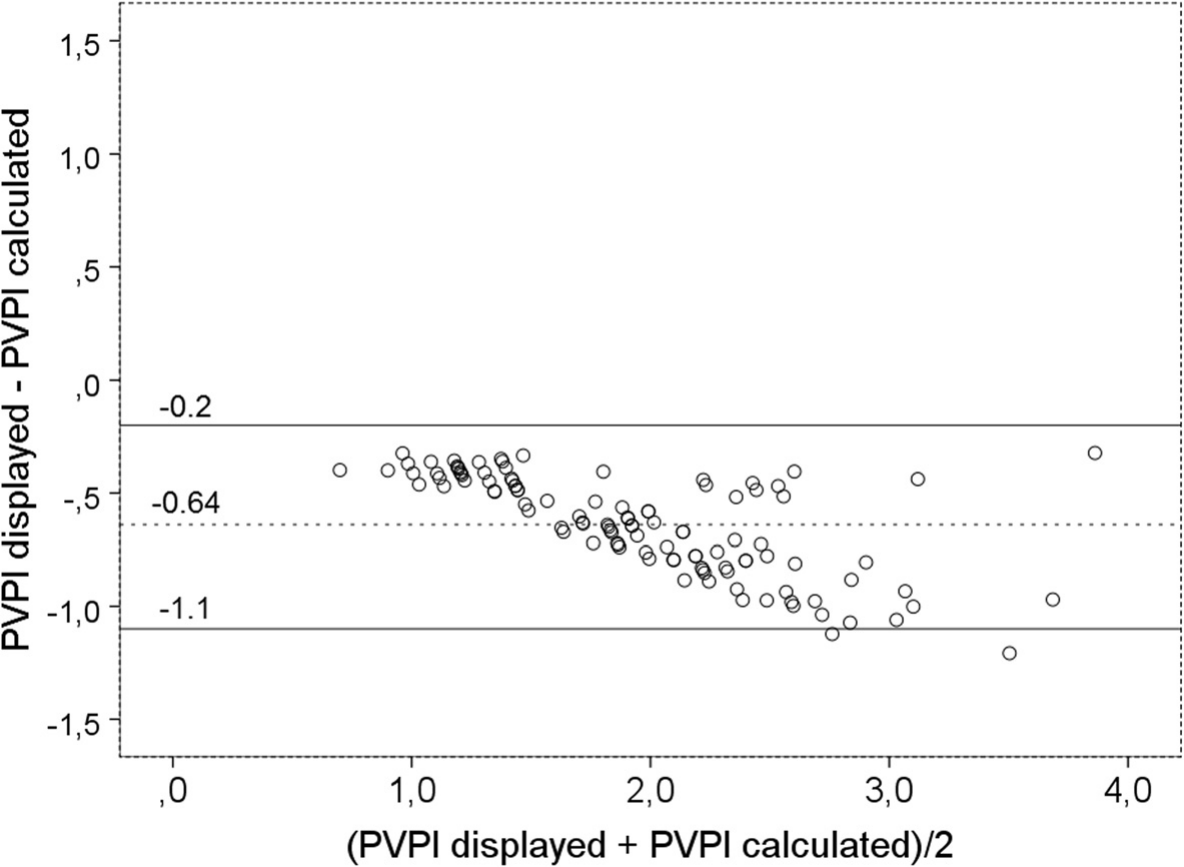
**Bland Altman plot comparing pulmonary vascular permeability index PVPI as displayed by the PiCCO-2 device (PVPI**_**displayed**_**) vs. PVPI calculated as ratio of EVLW/PBV (extravascular lung water/pulmonary blood volume).**

However, PVPI_displayed_ was significantly lower than PVPI_calculated_ (Figure 3; 1.64 ± 0.57 vs. 2.27 ± 0.72; p < 0.001).In addition to a bias of -0.64 ± 0.22 Bland-Altman analysis demonstrated a percentage error of 22% and upper and lower limits of agreement of -0.199 and -1.075 (Figure 4).

This could be explained if instead of the corrected GEDV_displayed_ an uncorrected GEDV_uncorrected_ was used for calculation of PBV and PVPI_displayed_.

Based on this hypothesis and also assuming that the formula for femoral indicator injection suggested by our group is used by the latest PiCCO algorithm we determined PVPI_corrected_ by multiplying PVPI_calculated_ by the ratio GEDV_uncorrected_/GEDV_displayed_.

**Figure 5:**
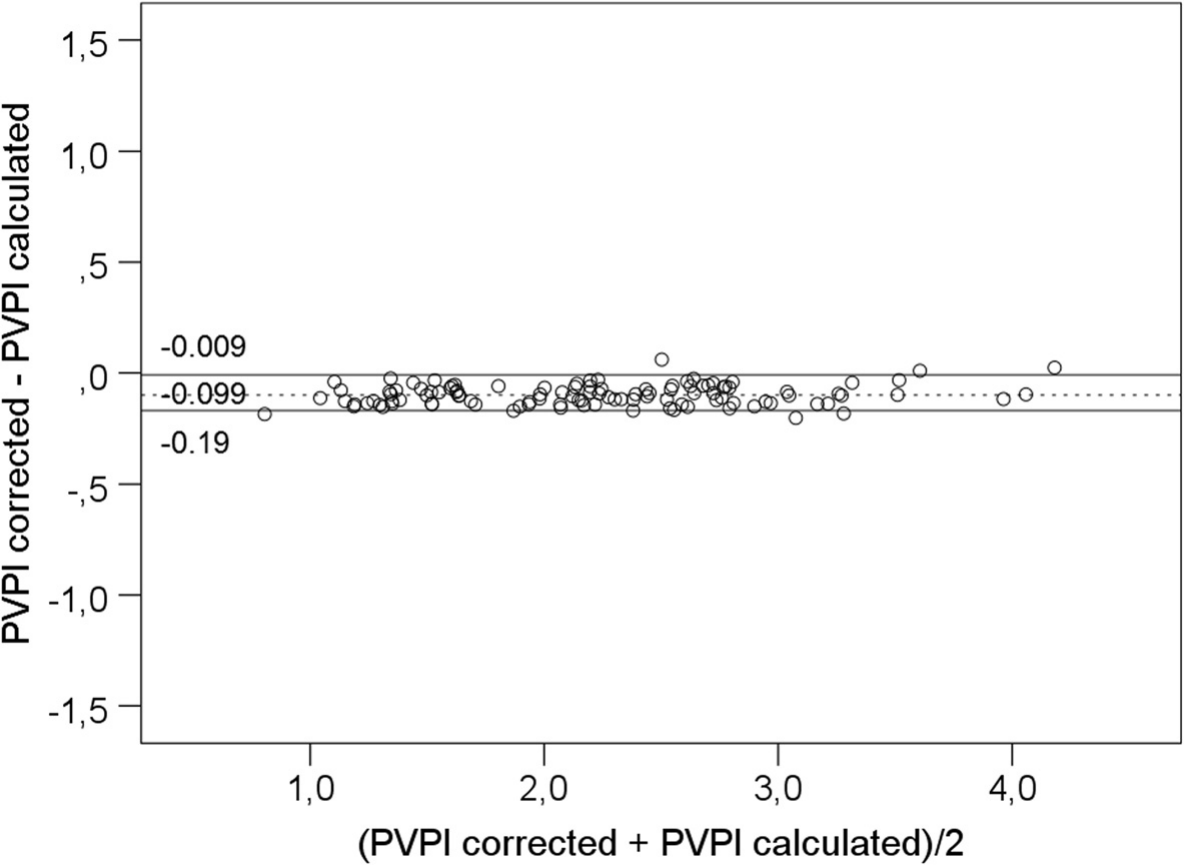
**Bland Altman plot comparing pulmonary vascular permeability index PVPI**_**displayed **_**corrected by the correction formula for femoral indicator injection (PVPI**_**corrected**_**; see Figure**3**) vs. PVPI calculated as ratio of EVLW/PBV (extravascular lung water/pulmonary blood volume).**

As shown in Figure 5 the bias of PVPI_displayed.d_ to EVLW/PBV could be reduced from -0.64 ± 0.22 to -0.10 ± 0.05. Furthermore, percentage error decreased from 22% to 4% with upper and lower limits of agreement of -0.009 and -0.190 (Figure 5).

## Discussion

Despite improving outcome by a number of specific and unspecific approaches such as low tidal volume [23], extracorporeal lung assist [24] and prone positioning [25] ARDS still carries a high mortality of up to 65% [23-26]. Well balanced fluid management providing appropriate resuscitation [27] as well as avoiding fluid overload-particularly of the lungs-is another obvious approach to improve outcome in ARDS. EVLW has been validated as a significant predictor of outcome in ARDS [3,7-11]. However, with regard to fluid management it is important, if increased EVLW derives from inflammatory capillary leakage of the lungs or from general fluid overload and congestive heart failure. Relating EVLW to pulmonary blood volume, PVPI has been demonstrated to discriminate inflammatory from congestive pulmonary failure [13,14]. Therefore, combined use of EVLW and PVPI has been suggested to specify therapy in acute pulmonary failure. However, there are a number of studies suggesting pitfalls in using these parameters derived from TPTD: Due to its current indexation solely to bodyweight EVLWI might be inappropriately lowered in obese patients. Therefore, weight correction formulas [19] and indexation of EVLWI to height [19,28] have been shown to improve usefulness of EVLWI. Furthermore, femoral central vein access for TPTD indicator injection inappropriately increases GEDV(I) [15,16]. Therefore, a correction formula for GEDVI in case of femoral CVC has been suggested [16]. Requirement of information about the CVC site in the last PiCCO algorithm suggests that some kind of correction for femoral CVC is provided. Due to the assumption that PBV is 25% of GEDV in single indicator TPTD [6,12], overestimation of GEDV in femoral indicator TPTD indicator injection also pertains to PVPI.

**Figure 6:**
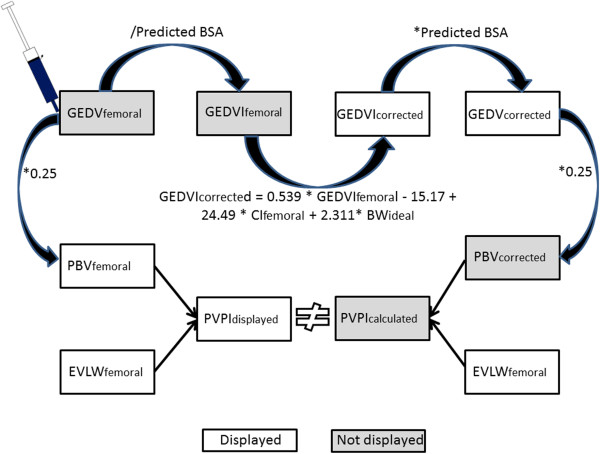
**Algorithm for the calculation of GEDV, GEDVI, PBV and PVPI as displayed by the PiCCO-2 software V 3.1 (PVPI**_**displayed**_**) and as suggested using GEDV**_**corrected **_**and PBV**_**corrected **_**for the calculation of PVPI**_**calculated**_**.** Values not displayed are shaded.

However, no information is available,

– if correction provided by the new PiCCO-algorithm applies only to GEDVI or also to GEDV

– if this correction is similar to the formula suggested by Saugel et al. [16] and

– if PVPI_displayed_ is based on PBV and GEDV derived from the correction formula suggested by our group.Therefore, we performed a prospective study in patients with femoral CVC access showing the following main results that are graphically summarized in Figure 6.

GEDV_displayed_ is corrected to the same degree as GEDVI_displayed_.

However, PVPI_displayed_ provided by the PiCCO-device is significantly lower than PVPI_calculated_ derived by dividing EVLW_displayed_ by 0.25*GEDV_displayed_. This suggests the use of uncorrected GEDV and uncorrected PBV for the PVPI displayed by the device.

This assumption is supported by PVPI_corrected_ values nearly identical to PVPI_calculated_ when correcting PVPI_displayed_ using the correction formula.

### Practical implications

Our data strongly suggest that the correction formula for GEDVI in case of femoral CVC indicator injection should be also applied for the calculation of PVPI. Otherwise, PVPI would remain to be artificially lowered in case of femoral CVC which also would result in different PVPI threshold for inflammatory vs. congestive pulmonary impairment.

### Limitations of the study

Despite conclusive results with high statistical significance this is a single centre study with a limited number of patients and TPTD measurements. Furthermore, we have to admit that our explanations for this inconsistency are in part speculative: Although correction of PVPI_displayed_ by our formula results in PVPI_corrected_ close to PVPI_displayed_, the hypothesis of a “neglect” of correction of PBV has to be proven by a confirmatory study. Future studies should verify the practical implications, e.g. showing improved association of PVPI with outcome when applying the correction formula also for PBV and PVPI. Furthermore, our findings could be confirmed by comparing PVPI derived from femoral and jugular indicator injection in patients with both femoral and jugular CVC.

Finally, these findings pertain to the PiCCO-2 device with the V3.1 algorithm. To the best of our knowledge, there is only one other device providing PVPI which is the Edwards EV 1000 VolumeView (Edwards Lifesciences; Irvine, CA, USA). Despite some minor modifications of the thermodilution analysis this device is comparable to the PiCCO. However, the EV-1000 does not require information about the CVC site. This suggests that the device does neither correct GEDV(I) nor PVPI in case of femoral CVC site.

## Conclusion

This study suggests that the V3.1 software of the PiCCO-device corrects GEDVI and GEDV for TPTD indicator injection based on a correction formula similar to the correction recently suggested [16]. However, correction of GEDV(I) does not pertain to PVPI which is contradictory to the definition of PVPI and results in a substantial underestimation of PVPI. To make PVPI values derived from jugular and femoral indicator injection comparable, corrected GEDV and PBV should be used for calculation of PVPI in case of femoral CVC.

## Abbreviations

APACHE-II: Acute physiology and chronic health evaluation-II; ARDS: Acute respiratory distress syndrome; BSA: Body surface area; CO: Cardiac output; CI: Cardiac index; CVC: Central venous catheter; EVLW: Extravascular lung water; EVLWI: Extravascular lung water index; GEDV: Global end-diastolic volume; GEDVI: Global end-diastolic volume index; ICU: Intensive care unit; ITTV: Intrathoracic thermovolume; PBV: Pulmonary blood volume; PE: Percentage error; PTV: Pulmonary thermovolume; PVPI: Pulmonary vascular permeability index; RRT: Renal replacement-therapy; ROC: Receiver operating characteristics; SOFA: Sequential organ failure assessment score; TPTD: Transpulmonary thermodilution.

## Competing interests

Wolfgang Huber collaborates with Pulsion Medical Systems Feldkirchen, Germany as member of the Medical Advisory Board. The other authors declare that they have no competing interest.

## Authors’ contributions

HB performed the majority of measurements, participated in analysis of the data and in drafting the manuscript and finally approved the manuscript. SM performed parts of the measurement, participated in analysis of the data and in drafting the manuscript and finally approved the manuscript. AB performed parts of the measurement, participated in analysis of the data and in drafting the manuscript and finally approved the manuscript. BH performed parts of the measurement, participated in analysis of the data and in drafting the manuscript and finally approved the manuscript. RS substantially contributed to conception and design of the study, participated in the analysis of the data, participated in drafting the manuscript and finally approved the manuscript. WH performed conception and design of the study, analyzed the data, drafted the manuscript and finally approved the manuscript.

## Pre-publication history

The pre-publication history for this paper can be accessed here:

http://www.biomedcentral.com/1471-2253/14/81/prepub
